# Incorporation of Silver Sulfadiazine into An Electrospun Composite of Polycaprolactone as An Antibacterial Scaffold for Wound Healing in Rats 

**DOI:** 10.22074/cellj.2020.6341

**Published:** 2019-07-31

**Authors:** Fereshteh Nejaddehbashi, Mahmoud Hashemitabar, Vahid Bayati, Eskandar Moghimipour, Jabraeel Movaffagh, Mahmoud Orazizadeh, Mohammadreza Abbaspour

**Affiliations:** 1 Cellular and Molecular Research Center, Ahvaz Jundishapur University of Medical Sciences, Ahvaz, Iran; 2 Department of Anatomical Sciences, School of Medicine, Ahvaz Jundishapur University of Medical Sciences, Ahvaz, Iran; 3 Nanotechnology Research Center, Faculty of Pharmacy, Ahvaz Jundishapur University of Medical Sciences, Ahvaz, Iran; 4 Targeted Drug Delivery Research Center, Pharmaceutical Technology Institute, Mashhad University of Medical Sciences, Mashhad, Iran

**Keywords:** Nanofibers, Polycaprolactone, Silver Sulfadiazine, Tissue Engineering, Wound Healing

## Abstract

**Objective:**

Fabrication of an antibiotic-loaded scaffold with controlled release properties for wound dressing is one of tissue engineering challenges. The aim of this study was to evaluate the wound-healing effectiveness of 500-µm thick polycaprolactone (PCL) nanofibrous mat containing silver sulfadiazine (SSD) as an antibacterial agent.

**Materials and Methods:**

In this experimental study, an electrospun membrane of PCL nanofibrous mat containing 0.3% weight SSD with 500 µm thickness, was prepared. Morphological and thermomechanical characteristics of nanofibers were evaluated. Drug content and drug release properties as well as the surface hydrophobicity of the nanofibrous membrane were determined. Antimicrobial properties and cellular viability of the scaffold were also examined. A full thickness wound of 400 mm^2^ was created in rats, to evaluate the wound-healing effects of PCL/SSD blend in comparison with PCL and vaseline gas used as the control group.

**Results:**

SSD at a concentration of 0.3% improved physicochemical properties of PCL. This concentration of SSD did not inhibit the attachment of human dermal fibroblasts (HDFs) to nanofibers *in vitro*, but showed antibacterial activity against Gram-positive *Staphylococcus aureus* (ST) and Gram-negative *Pseudomonas aeruginosa* (PS). Overall, results showed that SSD improves characteristics of PCL nanofibrous film and improves wound-healing process in one-week earlier compared to control.

**Conclusion:**

Cytotoxicity of SSD in fabricated nanofibrous mat is a critical challenge in designing an effective wound dressing that neutralizes cellular toxicity and improves antimicrobial activity. The PCL/SSD nanofibrous membrane with 500- µm thickness and 0.3% (w/v) SSD showed applicable characteristics as a wound dressing and it accelerated wound healing process *in vivo*.

## Introduction

The skin structure and function is often damaged by several factors such as chronic wounds, diabetic foot ulcers, surgical incisions, ruptures and burns. This unique tissue following exposure to these external threats, shows different reactions depending on the severity of the injury and the size of injured area (1). When over 1 cm of the skin is lost, skin grafting is necessary for avoiding bacterial infections, water and blood losses and extensive scar formation. 

As commonly used clinical approaches, autologous, allogenic and xenogeneic skin grafts have limitations such as availability of donor sites, risk of immune rejection and transmission of disease, respectively. Therefore, researchers are looking for ways to overcome these limitations. Various skin substitutes are used in the clinic, but none of them can restore the structure and function of the skin alone (2). 

Several studies have focused on tissue engineering methods using different types of biomaterials and electrospinning techniques (3, 4).

The important aim in this field is finding an ideal candidate for wound dressing. An acceptable biomaterial should present a panel of biomimetic characteristics such as extra cellular matrix (ECM) manifestations, biocompatibility, sustained release of drugs and reagents, very low cytotoxicity, wettability, biomechanical integrity, optimal biodegradability and anti- bacterial potency (5, 6). Polycaprolactone (PCL) is used in a broad spectrum of tissue engineering applications (7) and showed unique properties making it a good candidate for skin tissue engineering or wound dressing. The main advantage of PCL is its proper mechanical and handling characteristics, while the main drawback of PCL is its hydrophobicity that impedes the process of wound healing (8). To overcome this problem, and prepare a hydrophilic environment with antibacterial properties, silver sulfadiazine (SSD) as a hydrophilic antibacterial agent was added to PCL and a nanofibrous mat composed of PCL/SSD was fabricated (9).

SSD as a wide spectrum antibacterial and antifungal agent was used in formulation of burn ointments for several decades. It was shown that in addition to the anti-infective effects of SSD against a wide range of Gram-positive and negative bacteria, it promotes epithelialization and decreases inflammation and contraction of wound area (10, 11). However, the impact of incorporating SSD into nanofibrous mat, and its wound-healing properties have not been clarified yet. 

The aim of this study, at the first step, was to prepare and evaluate the efficiency of a cell-seeded nanofibrous mat of PCL comprising 0.3% w/v SSD. Then, a 400-mm^2^ wound was created in rats and fully covered by 500-µm thick PCL/SSD mats and the effectiveness of mats was evaluated. 

## Materials and Methods

### Study design

In this experimental study, SSD was incorporated into the PCL solution at the concentration of 0.3% and nanofibrous membrane of 500-µm thickness was produced using electrospinning technique. Then, main characteristics of the scaffold for transplantation in rats skin were evaluated. The rats were divided into the following 3 groups: rats treated with vaseline gas used as control group, rats treated with PCL/SSD, and rats treated PCL without SSD. 

### Materials 

Poly (ε-caprolactone Mw of 80KDa) (PCL), 3-(4,5-dimethylthiazol-2-yl)-2,5-diphenyltetrazolium bromide (MTT) (M2128), dialysis bag (12 KDa), triphenyltetrazolium chloride (T8877), Muller Hinton agar, and Muller Hinton broth (70192) were purchased from Sigma-Aldrich (USA). Acetic acid (purity 99.8%) was acquired from Merck (Germany). Fetal bovine serum (FBS), phosphate buffered saline (PBS), DMEM’F12, Trypsin, and penicillin/streptomycin (pen/strep) were purchased from Gibco (USA), and SSD was acquired from Sinadaru (Iran). 

### Methods

#### Preparation and characterization of polymeric solutions and nanofibers

The PCL pellets were dissolved in 90% acetic acid to produce 15% w/v polymer solutions; then, 3 mg/ml SSD was dissolved in the solution. The solutions were mixed using a magnetic stirrer overnight. 

In order to evaluate the influence of SSD on the rheological characteristics of PCL, the relative viscosity of the composite was assessed using Rheometer R/S plus Brookfield (Waukesha County, USA) at 20°C. 

The electrospinning of the composite was performed at 17 kV, flow rate 0.5 ml/hour, nozzle to collector distance 17 cm and drum rotation speed 125 rpm. 

Characterization of electrospun nanofibers was done under a field-emission microscope (Mira3Tescan, Czech), and prior to the examination, the samples were sputter coated with a thin layer of gold. Moreover, the SSD-loaded nanofibers were also characterized by energy-dispersive X-ray spectroscopy (EDX) (VEGA TESCAN, XMU, USA) to prove the presence of SSD in the nanofibers. 

#### Physicochemical characterization

##### Differential scanning calorimetry 

Differential scanning calorimetry (DSC) was carried out using STARe system (Mettler Toledo, Swiss). Then, 2 mg of the samples was heated in sealed aluminum pans under nitrogen flow (50 ml/minute) at a scanning rate of 10°C/minute from 25 to 300°C.

#### Thermogravimetric analysis 

To evaluate the thermal behavior of the samples, thermogravimetric analysis (TGA) was analyzed from room temperature to 600°C at heating rate 10°C/minute (STA503, Germany) under N_2_ flow. All the experiments were carried out in triplicate and the mean was reported. 

#### Mechanical test 

Tensile test was performed to evaluate mechanical characteristics of the mats. The scaffolds were cut in rectangular shapes (2×5 cm). Then, the film thickness was measured by thickness gauge and the tensile test was performed using a universal tensile machine (INSTRON 5967 USA) fitted with a 60 N load cell at 2 mm/minute speed until the samples were ruptured. 

#### Fourier transform infrared spectroscopy 

FTIR was performed to confirm presence or distribution of material in nanofibrous mat. The procedure was performed for PCL and SSD powders by mixing 50 mg samples with KBr and compressing to form pellets. The pellets were inserted into the FTIR spectrometer (Vertex 70 Bruker, Germany) connected to a PC and 30 scans with a resolution of 20 cm^-1^ were performed. For nanofibers, a sheet of nanofibers was detected and the data was analyzed using FTIR software.

#### Contact angle analysis 

Water contact angles of PCL/SSD and PCL nanofibrous membranes were measured by a water contact angle analyzer (FTA-125, First Ten Angstroms, USA). Samples (2×2 cm) were cut and placed on the testing plate; distilled water drops (3 µl) were used in all analyses.

#### Drug release studies

To determine the drug release rate, PCL/SSD nanofibrous mat (average weight 30 mg) was placed in the dialysis bag (cutoff 12,000 Da) with 5 ml PBS, immersed in 25 ml PBS (PH=7.4) in a 50-ml centrifuge tube and incubated at 37°C in a continuous horizontal shaker. At predetermined time-points, 2 ml of dissolution medium was retrieved and replenished with 2 ml of fresh PBS. Drug release profile was determined using UV absorption spectrophotometer (Shimadzu model uv-1700, USA) at 241 nm. 

#### Determination of PCL/SSD nanofibers degradation rate 

PCL/SSD matrices degradation rate evaluation was carried out in PBS (pH=7.2, at 37°C) in a shaking incubator for 7 days. Dry weight of matrices was measured on incubation days 1 and 17. Degradation was determined according to the following equation where w_0_ is initial weight, w is weight of matrix after degradation and w_1_ is degradation rate percentage. 

$$W_{1}(\%)=[\frac{W_{0}-W}{W_{0}}]\times 100$$

#### Antibacterial test

The minimum inhibitory concentration (MIC) of SSD on *Staphylococcus aureus* (ST) (ATCC29213) and *Pseudomonas aeruginosa* (PS) (ATCC27853) was briefly determined by using an antibiotic tube dilution method in supplemented Muller-Hinton Broth (12). The antibacterial properties of the PCL/SSD nanofibrous mat against ST (ATCC29213) and PS (ATCC27853) were briefly evaluated by zone inhibition test (13). 

#### Isolation of human dermal fibroblasts

Human skin specimens were obtained by plastic surgery (2×2 cm) from healthy individuals in compliance with a protocol approved by Ethics Committee of Ahvaz Jundishapur University of Medical Sciences (1394/657). The skin samples were kept in culture medium on the ice during transportation. The culture medium was composed of DMEM containing 0.5 µg/ml amphotericin B, 100 IU/ml gentamycin, 100 IU/ml penicillin and 100 µg/ ml streptomycin. The procedure of cell isolation was commenced as soon as possible in cell culture room of Cellular and Molecular Research Center (CMRC, Ahvaz Jundishapur University of Medical Sciences, Iran). The samples were sterilized in 70% ethanol for 10 seconds and rinsed 3 times with sterile PBS. The whole hypodermal adipose tissue and blood vessels were removed and discarded and cells were isolated according to a previously explained method (14). 

#### Cytotoxicity and cell adhesion studies 

For cytotoxicity studies, nanofibrous mats were punched and put onto 96-well culture plates. Human dermal fibroblasts (HDFs) were seeded at 5×10^3^ cells per well on both PCL/SSD, and PCL nanofibrous mat. MTT assays were performed on days 1, 3, 6, and 9 using a microplate reader (Bio-Rad 680, USA) at 570 nm. 

The cell adhesion studies on the PCL/SSD nanofibers were carried out using HDFs after 24 hours. Electrospun nanofibrous mats were sterilized by 1-hour UV radiation done prior to cell studies. Cells were added to each nanofibrous mat at a seeding density of 10^4^ cells/cm mat. After 24 hours, the fibers were washed thrice with PBS, then fixed using 2.5% glutaraldehyde for 1 hour at 4°C, dehydrated by graded ethanol and allowed to air-dry overnight. The dried samples were imaged using field emission-scanning electron microscope (FE-SEM). 

#### *In vivo* evaluation

##### Creation of full thickness wound

Fifty-four male Sprague-Dawley rats (250 g) were housed under standard conditions at controlled temperature (21 ± 2°C) with 12/12 hour light/dark cycles. All protocols were done according to the Ethics Committee of Ahvaz Jundishapur University of Medical Sciences (1394/657). Animals were anesthetized by 40 mg/kg ketamine and 5 mg/ kg xylazine; then, dorsal surface was shaved by an electric hair clipper and sterilized using 10% povidone-iodine. A full thickness square wound (400 mm^2^) was cut with a scissor from the back along the dorsal side of the skin of each rat. The scaffolds were affixed using 5-0 nylon sutures. One wound was created on each rat and 18 rats were used in each group but some of them were lost during anesthesia. The following three groups were used in this study: control group treated with vaseline gas (n=12), PCL/SSD-treated group (n=12) and PCL nanofibrous mats-treated group (n=12). Considering the mean thickness of nanofibrous mat, approximately 500-µm thick scaffolds were applied. On days 14, 21, and 28, animals were euthanized, and the process of wound closure was observed by using a digital camera, and then the surrounding skin and muscle including wound area were removed, fixed in formalin and embedded in paraffin. 

### Histological analysis

The reconstituted wound region of all groups was removed to the level of hypoderm layer. The specimens were fixed in 10% formaldehyde. Samples were cut into 2-µm thick sections by a rotating microtome for histological studies and for evaluation of wound area and repairing process. Hematoxylin and eosin (H&E) and Masson’s trichrome staining were performed.

###  Statistical analysis

The statistical analysis was performed using the SPSS for windows, version 16 (SPSS Inc., IL, USA). The difference in means of the continuous data was evaluated using one-way analysis of variance (ANOVA) followed by Tukey post hoc analysis. All experimental data were presented as mean ± SEM. Each experiment was repeated at least 3 times. A P<0.05 was considered statistically significant. 

## Results

### Solutions and nanofibers characterization 

**Fig.1:**
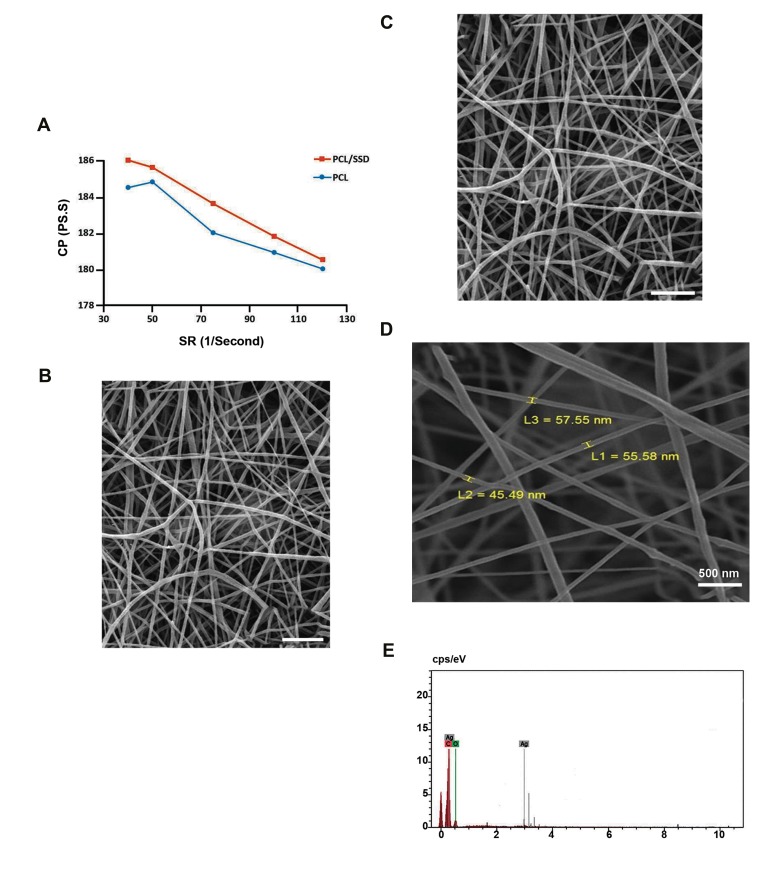
Solution and fiber characterization. **A.** Rheology test for PCL and PCL/SSD mats, **B.** Field emission electron microscopy (FE-SEM) for PCL/ SSD mat (scale bar: 2 µm), **C.** PCL mat (scale bar: 2 µm), **D.** Scanning electron microscopy imaging, and E. Energy dispersive spectra of PCL containing 0.3% SSD. PCL; Polycaprolactone, SSD; Silver sulfadiazine, CP; Centipoise, SR; Shear rate, and L 1, 2, 3; Size 1, 2, 3.

The viscosity (.) of PCL solutions in the presence and absence of SSD, at different shear rates (SR), are shown in Figure 1A Compared to 15% wt PCL alone, the viscosity of composite of 15% PCL and 0.3% weight/volume SSD was slightly increased. The viscosity of both solutions decreased due to increase in SR, which indicated a non- Newtonian type of fluid. For both PCL and PCL/SSD, SR were in the range of 40-120 second^-1^, while the viscosity was in a range of 180-184 pa.s for PCL and 181-186 pa.s for PCL/SSD. Thus SSD increased viscosity of the composite, but did not affect the integrity of nanofibers.

FE-SEM images of electrospun PCL and PCL/SSD mats showed uniform and beadles nanofibers (Fig .1B, C). Mean diameter for PCL and PCL/SSD was 116.82 and 218.62 nm, respectively. Therefore, SSD incorporated uniformly in solution and nanofibrous mat. 

EDX evaluations are shown in Figure 1D and E and the peak of Ag shown in the Figure 1E the presence of SSD in PCL nanofibrous mat. Elementary analysis of nanofibers was carried out by using SEM-EDX (Fig .1D). Carbon and oxygen as the main elements presented in the PCL nanofibers, and also that of silver as a marker of SSD agent, were detected (Fig .1E).

### Physicochemical characterization 

#### Differential scanning calorimetry analysis 

**Fig.2:**
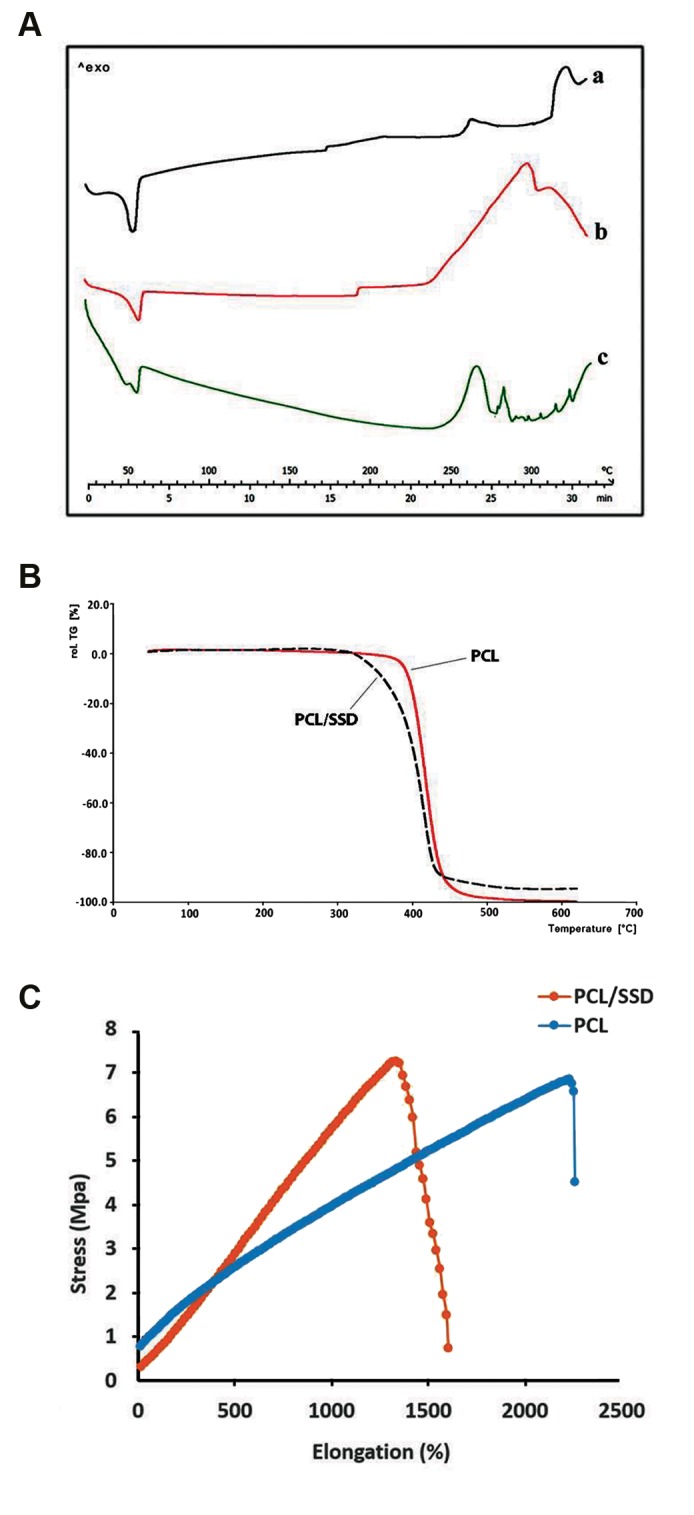
Physicochemical characterization. **A.** DSC thermogram for (a) PCL/ SSD nanofibrous mat, for (b) PCL nanofibrous mat, and (c) physical mixingof PCL and SSD, **B.** TGA results for PCL and PCL/SSD nanofibrous mat, and **C.** Mechanical behavior of the mats with and without SSD. DSC; Differentialscanning calorimetry, PCL; Polycaprolactone, SSD; Silver sulfadiazine, and TGA; Thermogravimetric analysis.

In DSC thermogram, the endothermic melting peak for PCL nanofiber appeared at 60.06°C and for physical mixture of PCL and SSD was 60°C, while for PCL/SSD nanofibrous mat was 56.6°C. This shift in melting point from 60 to 56.6°C could be attributed to the interaction between drug and polymer during electrospinning process and changes in their physical structure Figure 2A

### Thermogravimetric analysis 

TGA curves of PCL, SSD and PCL/SSD nanofiber are presented in Figure 2B The initial decomposition temperature for PCL was around 300°C, for SSD was 288.7°C, and for PCL/SSD was around 300°C. 

### Tensile test

The Young’s modulus of PCL and PCL/SSD nanofibrous membranes were 1.3 and 0.65 MPa, respectively (Fig .2C). These results were in the range of elastic modulus in normal human skin (i.e. 0.2-20 MPa) indicating acceptable mechanical strength and elasticity for both nanofibrous mats (15). 

### Fourier Transform Infrared spectroscopy analysis

**Fig.3:**
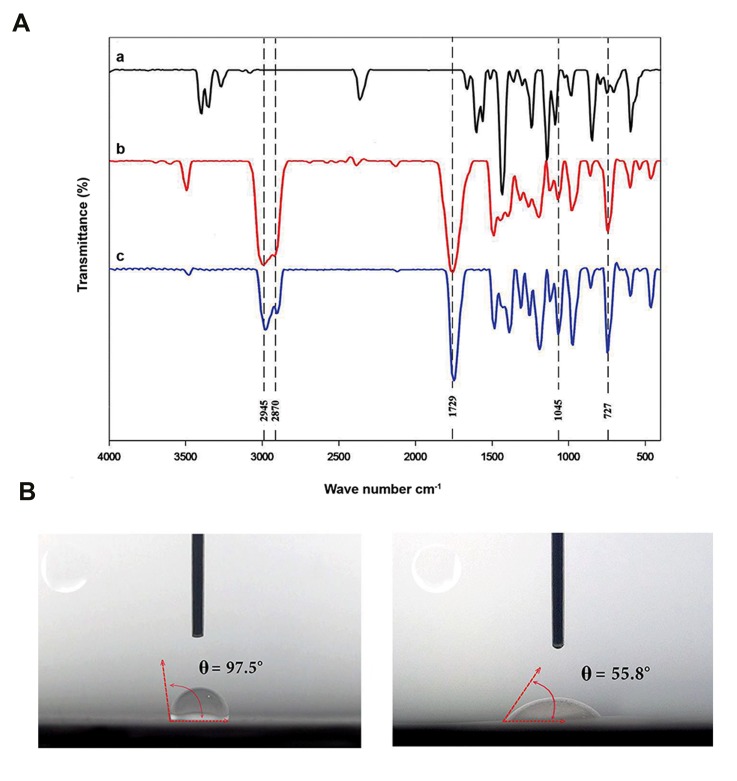
FTIR and contact angle for nanofibrous mat. **A.** FTIR for (a) SSD, (b) PCL, and (c) PCL/SSD nanofibrous mat are shown, **B.** Contact angle for PCL, and **C.** PCL/SSD are shown. FTIR; Fourier Transform Infrared spectroscopy analysis, SSD; Silver sulfadiazine, and PCL; Polycaprolac.

FTIR spectrum for SSD, PCL and PCL/SSD nanofibrous mats are shown in Figure 3A FTIR spectrum of the PCL exhibited characteristic peaks at 2945.91 cm^-1^ (CH2-asymmetric and stretching), 2870.75 cm^-1^ (CH2-symmetric stretching) and 1729.26 cm^-1^ (C=O-stretching). The chemical structure of the PCL/SSD nanofibrous mats was evaluated by FTIR to examine chemical interactions between the PCL and SSD, as shown in Figure 3A Moreover, PCL/SSD nanofibrous mats showed additional bands at approximately 1045.95, and 727.84 cm^-1^, which are representative of various vibration modes of N-C, N-O bonds. The broad peak observed at 3500 cm^-1^ might be due to the hydrogen bond interaction between PCL and SSD. 

### Contact angle test 

Contact angle of nanofibers was 97 ± 2° and 56 ± 2° for PCL and PCL/SSD nanofibers, respectively Figure 3B, C These measurements showed that incorporation of SSD into PCL nanofibers leads to higher hydrophilic surface of the nanofibrous membrane. 

### Drug release and antibacterial effects

**Fig.4:**
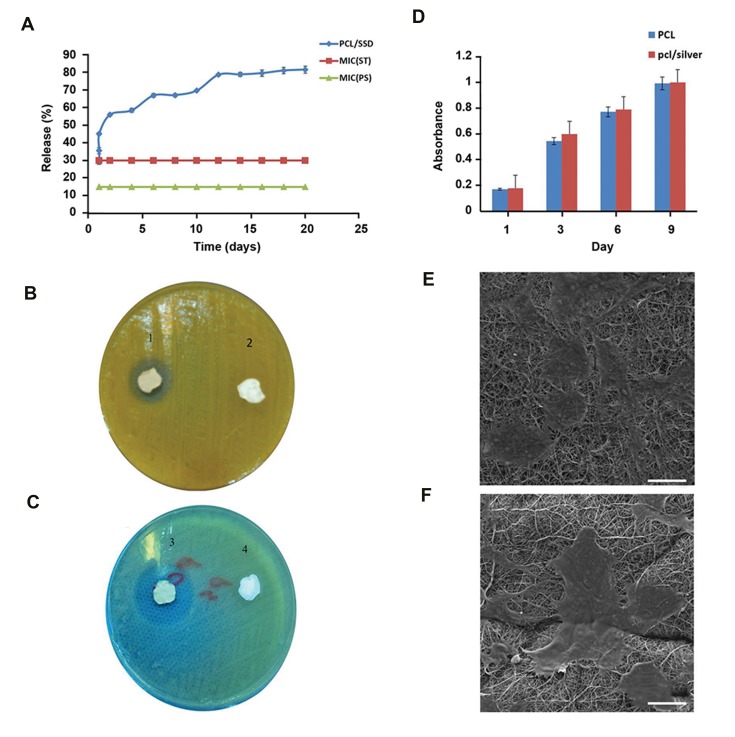
Drug release profile. **A.** *In vitro* SSD release from PCL/SSD nanofibrous mat, **B.** Antibacterial test for PCL/SSD, 1 and 3 are PCL mat containing SSD, and 2 and 4 are PCL mat. *B. is Staphylococcus aureus* and **C.** is *Pseudomonas aeruginosa.* MTT assay for PCL and PCL/SSD nanofibrous mat, **D.** Differences between two nanofibrous mat were not significant, **E.** Attachment of cells on PCL nanofibrous mat (scale bar: 50 µm), and **F.** PCL/SSD nanofibrous mat are shown (scale bar: 50 µm). SSD; Silver sulfadiazine, PCL; Polycaprolactone, and MTT; 3-(4,5-dimethylthiazol-2-yl)-2,5-diphenyltetrazolium bromide.

The cumulative release profile of SSD from the nanofibrous mat is shown in Figure 4A The profile exhibits an almost fast release of the drug (up to 60%) in 4 days followed by a sustained release of 80% drug during 20 days. It was shown that PCL could modulate the release profile of anti-infection reagent (12). Antibacterial properties of scaffold were assessed against *Staphylococcus aureus* (ST, ATCC 29213) and *Pseudomonas aeruginosa* (PS, ATCC 27853) using inhibition zone measurements. Clear inhibition areas around the samples containing SSD affirm their antibacterial properties (Fig .4B, C). Considering the MIC for *Pseudomonas aeruginosa* (15 µg/ml) and *Staphylococcus aureus* (30 µg/ml), it can be concluded that the concentration of drug released over a period of 20 days, was above the MIC of the microorganisms. Moreover, the release of 50% drug within the first 3 days has merits for fast effectiveness of the nanofibrous mats. 

### Biodegradability

Among many classes of biodegradable and biocompatible polymers, PCL is a suitable polymer for producing nanofibers by electrospinning. Slow degradation and release rate of this polymer is an advantage for its application in drug delivery systems,and PCL shows little degradation in an aqueous environment (16). 

Biodegradability for PCL/SSD nanofibrous mats is an important parameter that shows controlled release of SSD during incubation days. In this way, we digested 3 mg of the scaffold using a 90% acetic acid as solvent at once, measured the absorbance of the solution, and plotted the standard curve for different concentrations of the drug. Absorption of 0.19 is equivalent to 3 µg of the drug that is present in 3 mg of scaffold. After incubation of 30 mg scaffold for 7 days, approximately 30 µg of drug were released. Therefore, the degradation rate was 0.1%. 

### Cytotoxicity and cell attachment studies

MTT assay was done on days 1, 3, 6, and 9 to study the toxic effects of SSD incorporated into nanofibers and evaluate the biocompatibility of mats (Fig .4D). In order to evaluate cell adhesion and spreading on PCL/ SSD nanofibrous mat, HDFs cells were seeded in PCL/ SSD mat for a period of 24 hours. FE-SEM images (Fig .4E, F) showed cell adhesion on this nanofibrous mat. Cell proliferation and cell adhesion were obviously observed when cultured on both nanofibers. Due to hydrophilicity of PCL/SSD nanofibrous mat, cells attachment and proliferation rates were clearly higher than PCL nanofibrous mat (Fig .4E, F). 

### Macroscopic evaluation 

**Fig.5:**
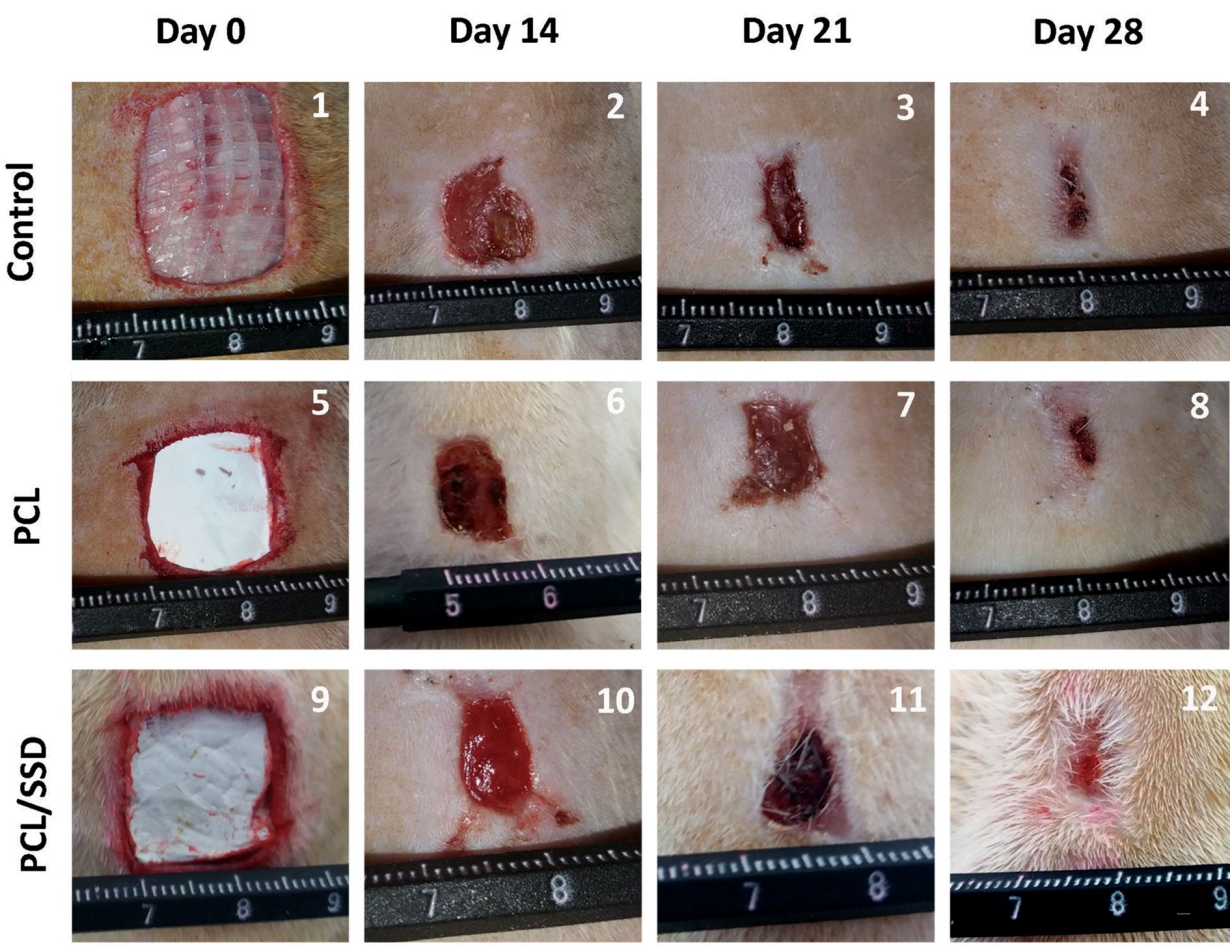
Macroscopic observation. The process of wound closure during the healing time was evaluated for assessment of PCL and PCL/SSD nanofibrous mat. PCL; Polycaprolactone and SSD; Silver sulfadiazine.

The appearance of each wound was observed on days 14, 21, and 28 of treatment. As shown in PCL/SSD group, wound healing process was remarkable compared to PCL and control after 14, 21 and 28 days. The extent of wound healing was evaluated by comparing wound size at each time-point with the primary wound size on day 0 (Fig .5) (1, 5, 9). Electrospun PCL/SSD mat and PCL alone were placed and adhered on the wound site of the test groups. From day 14 to 28, the PCL/SSD nanofibrous mat showed faster contraction compared to both open wound samples as control and PCL samples. On day 21 of treatment (Fig .5) (3, 7, 11) scab fell off the skin wound in all samples, but PCL/SSD sample showed a faster healing with more regenerated skin and more hair growth. On day 28 of treatment (Fig .5) (4, 8, 12), all wounds appeared to be closed, and scab was observed upon open wound in control and PCL group. 

The wound size in different groups was measured; in PCL/SSD group, more than 80% wound closure was found by day 14, while in PCL group, about 40% wound closure was observed. In PCL/SSD group, wound closure finalized (~90%) until day 21, while in PCL group and control group it reached 60 and 40%, respectively on day 21. Thus, PCL/SSD as wound dressing accelerated the healing process and shortened the final healing time to 14 days compared to 21 and 28 days for PCL group and open wound, respectively. 

### Microscopic and histological evaluation

**Fig.6:**
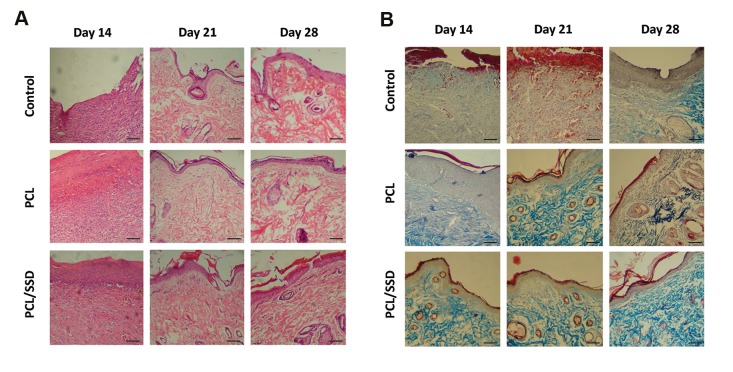
Microscopic observation: histological evaluations of wounds treated with PCL, PCL/SSD and control, on days 14, 21, and 28 of the healing process are shown. **A.** H&E staining and **B.** Masson’s trichrome staining (scale bar: 50 µm).

To evaluate the *in vivo* efficacy of nanofibrous mat, H&E staining was done on sectioned tissue samples obtained from the wound site. The results for H&E staining on days14, 21, and 28 post-wounding were summarized in Figure 6A Histological studies showed accelerated healing time for PCL/SSD used as a wound dressing. 

On day 14, in control group, thick granulation tissue with inflammatory cells including lymphocytes and neutrophils, obviously occupied wound site. However, in PCL group, the thickness and density of granulation tissues was clearly decreased and distribution of inflammatory cells was moderate. The granulation tissue and related inflammatory cells in PCL/SSD group were decreasing, but, fibroblast-like cells were increased and the repair process was promoting. 

On day 21, both test groups showed noticeable improvements in regeneration of epidermis and dermis. Proliferation of fibroblasts and reformation of collagen fibers were observed. All these changes were observed in PCL group but in at a moderate level compared to test groups, in control group, in epidermis and dermis, the tissue characteristics were similar to a skin in primary stages of regeneration and no blood vessels and collagen formation were clearly found. On day 21, epithelialization showed perfect thickness and morphology in PCL/SSD group compared to PCL and control group (Fig .6A). Typical vascular morphology, normal and desirable format of collagen fibers and bundles, were clearly observed. Thus, on day 21, in PCL/SSD group, the healing process was fully completed. But, in PCL group, the epithelialization, vascularization and collagen forming showed undesirable results. Therefore, by using the PCL/SSD as a wound dressing, healing time was accelerated, and the process moved to an acceptable and optimal route, to produce a normal skin with high quality and proper appearance. In other words, the process of healing was completed during 14 days and after that, the appearance and morphology was similar to a final perfect manifestation of the normal skin. 

Overall, in histological analysis, inflammatory cells, fibroblasts and fibroid debris were seen in control group until day 21, but these features were seen in PCL/SSD and PCL nanofibrous mat group until day 14. 

On day 28, all these groups showed final steps of regeneration and skin formation. These alterations in PCL/SSD showed very clear and developed stages of skin reformation toward a normal skin. Epithelialization and also skin appendages like hair follicles and sebaceous glands in PCL/SSD were obviously visible. Although in the two other groups, these presentations were observed, but, these changes were similar to PCL/SSD group on day 14. In other words, the process of regeneration was clearly shortened for about one week. The shape of wounds covered with PCL/SSD and PCL was rounded whereas the open wound was elongated, due to contraction of the rat skin. The results showed significantly reduced scar at both macroscopic and histological levels in the PCL/SSD mat compared with PCL mat and open wound, on day 28. 

Masson’s trichrome staining (Fig .6B) was used for evaluation of collagen formation and remodeling presented very sharp difference among PCL/SSD and other groups. In control group, collagen formation was in early steps and maturation of fibers and bundle formation was not occurred properly. In PCL group, when compared with control, the development of collagen bundles was promoted but compared to PCL/ SSD group, the process of collagen formation was in earlier stages. Compared to the other groups, the PCL/ SSD group showed the final and developed collagen bundles with desirable morphology which was similar to normal skin. This morphology and arrangement of collagen bundles in PCL/SSD clearly showed the production of a high-quality skin that could function as a perfect skin. 

On day 21, when different groups were compared, the skin overview of PCL group was similar to that of PCL/ SSD group on day 14.

In other words, the PCL group with about one-week delay showed presentations similar to those of the PCL/ SSD group. Development of dermis and collagen bundles in PCL/SSD group were also observed. On day 28, different groups showed progressive phase of repairing, but in PCL/SSD group, final skin remodeling in dermis layer was visible and PCL group still needed more time to produce the final form of a normal skin. 

## Discussion

As a wound dressing, a proper biomaterial with optimized characteristics should be able to decrease inflammation, infection, and scar formation and promote normal skin remodeling. Moreover, it should possess a proper surface for cell attachment and proliferation. Loading of drug into nanofibrous mat is a way to deliver drug into target region in a sustained manner. Nanofibrous mats with similar characteristics to those of ECM, are desirable candidates to be used as an optimum wound dressing and possess an acceptable potential for loading antibiotics to decrease the risk of infection (17, 18). 

This study presents three main findings as follows: i. Design and fabrication of nanofibrous mat containing effective concentrations of SSD against *Staphylococcus aureus* and *Pseudomonas aeruginosa* that were approved *in vitro*, ii. Construction of a nanofibrous mat with 500 µm thickness, similar to wound thickness, as a wound dressing, and iii. Creation of an expanded wound area (400 mm^2^) compared to similar studies.

In this study, functionality of optimized nanocomposite PCL containing 0.3% SSD was evaluated in treatments of full area and thickness (400 mm<sup>2</sup> and 500 µm, respectively) wound healing in wistar rats and healing process were compared to that of rats treated with PCL nanofibers and vaseline gas used as control group.

Results of the present study showed that the PCLsolution containing 0.3% SSD, has suitable viscosity and it was successfully synthesized by electrospinning in the range of nanoscale fibers. This concentration of SSD showed effective antibacterial properties *in vitro* and a controlled release profile for SSD during 20 days. Physicochemical and mechanical analysis such as TGA, DSC, and contact angle showed suitable characteristics of this scaffold in regeneration of skin tissue.

For several decades, SSD has been used as an antibiotic for wound healing. However, there are few studies that focused on nanofibers containing SSD. Using electrospun mats containing SSD is a promoting method to fabricate antimicrobial wound dressings. For this reason, it is necessary to evaluate the physical and antimicrobial features as well as the biocompatibility of nanofibers loaded with SSD, to optimize the concentration of SSD.

The proper physical and mechanical integrity of the nanofibrous mat and similar viscoelasticity and flexibility to skin tissue, are the main characteristics of a desirable mat designed for wound dressing applications (1, 19). Therefore, in the first step to obtain an economic, nontoxic, and also an optimum backbone of scaffold, the unique solvent system (90% acetic acid) was selected as solvent (20, 21) for dissolving PCL. In addition, a very low but effective and nontoxic concentration of SSD (0.3%) was incorporated into the polymeric solution (22, 23). Fabricated PCL/SSD nanofibers showed acceptable characteristics with respect to biocompatibility, drug loading potential, anti - infection properties, and improved wettability to promote cell attachment, which play crucial roles in wound healing.

Some studies showed that modification of solutions viscosity affects the nanofiber characteristics. Some other works used different active ingredients such as plant extract, silver nanoparticles and Ag ions to increase the hydrophilicity and antimicrobial activity of the scaffold (24, 25). According to our results, the diameter of nanofibers in mats and also the elasticity of their composite showed an optimum range in nanoscale dimensions. In our study, by addition of SSD, viscosity of PCL/SSD composite was increased, and uniform and beadless nanofibers with a continuous, uniform and randomly-oriented morphology was formed. 

Contact angle and tensile test showed that incorporation of SSD results in weaker mechanical properties and more hydrophilic surfaces of PCL nanofibers. It was shown that a hydrophilic surface leads to higher affinity for cell interactions (22). MTT and FE-SEM analysis of scaffold with HDFs and their ability for cellular attachment, also showed that cellular proliferation pattern with applied concentration of SSD (0.3% wt) did not significantly alter PCL nanofibers integrity and cell tolerability. 

SSD release profile showed that the amount of SSD released from the nanofibers was above the MIC for *Pseudomonas aeruginosa* and *Staphylococcus aureus* even at the very starting time-points. 

In a previous study, Mohseni et al. (19) studied the effect of three different concentrations of SSD on the characteristics of PVA/PCL nanofibers *in vitro*; but, the study did not evaluate the *in vivo* effect of SSD-loaded nanofibers on wound healing. In another *in vitro* study, Mim et al. showed that Ag/PCL/Ge nanofibrous can protect wounds from bacterial infection and promote tissue regeneration, but they did not perform *in vivo* studies for evaluation of the effect of this scaffold on wound healing process (26).

In this study, PCL nanofibrous mat containing SSD was fabricated and applied *in vitro* and *in vivo*. PCL is a nontoxic polymer that is commonly used in tissue engineering because of its great biocompatibility and biodegradability characteristics, as well as its ability to provide a sustained release of anti-infection agents.

Some previous studies used higher concentrations of SSD such as 1, 2, and 3% (22), it was shown that using a silk biomaterial treated by dipping in a mixture solution of EGF and SSD for 48 hours at 4 °C, can improve the wound healing (26).

Semnani et al. (27) impregnated SSD into PVP/gelatin scaffold by electrospining; this membrane showed antibacterial activities against Gram-negative and -positive bacteria *in vitro*. However, the *in vivo* study was not performed for this scaffold. In this study, different ratios of loaded drug (0.1, 0.2, and 0.3 %) were tested and it was shown that samples with 0.3% drug had higher drug release rate and in turn, a greater antibacterial activity.

In the final step of our study, we applied the nanofibrous mats as a wound dressing on animal wounds and followed the healing process for 14, 21, and 28 days. Histological staining of repaired tissue in PCL/SSD group after 28 days did not show scarring in wound area in comparison to control. Jin et al. (28) created a wound size of around 8 mm^2^ on mice and after 20 days, the wound was not closed completely. But, in our study, a wound size of about 400mm2 was created on the back of rats, and after 28 days, the wound was completely closed in PCL/SSD groups. Jeong et al. (22) designed a silk fibroin nanofibers containing 0.5, 0.1, and 1 % wt SSD that 1% wt SSD inhibited the attachment of epidermal cells to SF nanofibers *in vitro* and were cytotoxic to attachment of normal human epidermal keratinocyte (NHEK) and normal human epidermal fibroblast (NHEF). A6-mm diameter biopsy punch was created on the dorsum of the rats and after 15 days, the healing process was comparable to control group.

In the study of jasmine stojkovska in 2018, silver nanoparticles accelerated the healing process of a thermal burn model with 10 mm in diameter between 19-21 days, but in our study, full thickness wound with 20 mm in diameter repaired after 21 days in PCL/SSD nanofibrous mat with 500-µm thickness. In this group, dermis and epidermis and collagen bundles with normal features similar to normal tissue were seen (29). 

Comparing the regeneration stages and reformation of wounded skin, obviously presented this idea that by using PCL/SSD electrospun mat, wound healing accelerated for about one week. In other words, PCL/SSD as a wound dressing could finalize the regeneration and reformation by the end of week two. When this macroscopic presentation was examined by histological assessment, epidermis and hypodermis showed a reformed and remodeled skin. A favorite reepitelialization, rearrangement the collagen fibers and bundles similar to normal skin by specific staining, and also formation and dispersing of blood vessels in dermis were the main alterations that were clearly observed.

The main strength and novel points in this study are application of a mat with 500 µm - thickness in vivo on a larger wound area (about 400 mm^2^). A thickness of 500 µm nanofibrous mat is similar to epidermis plus dermis thickness in normal skin. Since the smaller wounds are healed in shorter time points, we applied a larger wound area in our study, to examine the effectiveness of PCL/ SSD mat.

It seems that application of PCL with 0.3% SSD and 500 µm -thickness could accelerate wound healing in about one-week shorter period. Specific staining for collagen showed thick collagen bundles in dermal layer in PCL/SSD group compared to PCL and control group. Additionally, the rate of epithelialization and formation of skin appendages such as new hair follicles and sebaceous glands were higher in PCL/SSD group compared with PCL and control. Moreover, these findings are supported by the previous studies (29-31).

## Conclusion

This study demonstrated that PCL/SSD blended mat could be considered a wound dressing for fast and effective repairing and remodeling of skin tissue. Further studies are needed to assess the effect of PCL/SSD nanofibrous mat containing higher concentrations of SSD during a short period of wound healing to accelerate the healing process. 
